# Human calprotectin as a novel biomarker in multiple sclerosis: Can it differentiate disease stages?

**DOI:** 10.1097/MD.0000000000046677

**Published:** 2026-01-30

**Authors:** Elif Banu Soker, Miray Erdem, Derya Ozdogru, Yusuf Tamam

**Affiliations:** a Department of Neurology, University of Health Sciences, Adana City Training and Research Hospital, Adana, Turkey.

**Keywords:** disease-modifying therapies, human calprotectin, multiple sclerosis, progressive multiple sclerosis, relapsing-remitting multiple sclerosis

## Abstract

This study aims to investigate the relationship between serum calprotectin levels and different stages of multiple sclerosis (MS). This study was designed as a prospective, cross-sectional case-control study. A total of 98 patients diagnosed with relapsing-remitting MS and secondary progressive MS according to the 2017 McDonald criteria, along with 40 control patients whose human calprotectin levels were measured, were included in the study. The average age and human calprotectin levels in the patient group were found to be higher compared to the control group (*P* = .013 and *P* < .001, respectively). No significant differences were observed between groups for other parameters (*P* > .05). A higher frequency of the relapsing-remitting MS type was observed in patients with a high calprotectin cutoff value within the patient group (*P* = .035). Our study suggests the potential of serum calprotectin as a valuable biomarker in MS, both in aiding diagnosis and potentially distinguishing MS subtypes.

## 1. Introduction

Multiple sclerosis (MS) is an autoimmune disease primarily affecting the central nervous system (CNS) and characterized by demyelinating changes and inflammation.^[1,2]^ MS is the most common neurological disease among young adults globally and typically diagnosed during this period. While the characteristic features of MS can be observed across all disease stages, the pathological processes, disease progression and activity can vary between relapsing-remitting MS (RRMS) and progressive MS, including primary progressive MS (PPMS). The pathophysiology of MS begins with an aberrant immune response attacking nerve cells and destroying the myelin sheath, leading to impaired nerve transmission. During the progression of MS, demyelination and inflammation are pronounced in early stages, while degeneration and neurological function loss become prominent in later stages.^[2]^

Current therapeutic strategies for MS primarily aim to reduce relapse frequency, slow disease progression, and manage symptoms. Disease-modifying therapies (DMTs) are the cornerstone of MS treatment. Injectable DMTs, such as interferon beta and glatiramer acetate, have been available for many years and are generally well-tolerated, but their efficacy can be limited for highly active disease, and they require frequent self-administration. Oral DMTs, such as fingolimod and teriflunomide, offer convenience but may cause systemic side effects. Infusible DMTs, particularly natalizumab, alemtuzumab, and ocrelizumab, are considered effective in reducing relapses and disability progression. However, these therapies necessitate careful patient monitoring since they are associated with more significant risks, including secondary autoimmune disorders or infusion reactions. The choice of DMT depends on factors including disease activity, patient comorbidities, and individual risk tolerance.^[3]^

Human calprotectin (Hcal) is a calcium-binding protein that is defined as a heterodimer of S100A8 and S100A9 (S100A8/A9) protein families. Hcal is known to be involved in a wide range of physiological processes. It is produced by granulocytes that differentiate into monocytes and microglia and are released through activated endothelial-monocyte interaction.^[4]^ Fecal calprotectin levels are routinely utilized as an inflammatory biomarker in the diagnosis and follow-up of inflammatory bowel diseases (IBD).^[5]^ Increased serum calprotectin levels are also highlighted in other conditions where inflammation is prominent, including cystic fibrosis, rheumatoid arthritis, and systemic infections.^[6–8]^

Studies on calprotectin measurement in MS patients suggest that cerebrospinal fluid (CSF) calprotectin levels may reflect disease activity in MS.^[4]^ Although it has been suggested that an increase in serum calprotectin levels may play a role in disease activity in MS patients, more studies are needed to support these findings. Furthermore, literature research shows there are no studies regarding whether calprotectin levels are an effective biomarker in differentiating MS subgroups. Clinical follow-up of MS is crucial for monitoring transitions between disease stages, assessing treatment responses and evaluating prognosis. Therefore, we aimed to investigate the relationship between serum calprotectin levels and different stages of MS in our study.

## 2. Methods

### 2.1. Study design and data collection

This study was designed as a prospective, cross-sectional case-control study. Ethical approval was obtained from the Ethics Committee of Health Sciences University, Adana City Training and Research Hospital, on March 14, 2024, with protocol number 3217. All participating patients provided informed consent. The study was conducted between April 1, 2024, and June 31, 2024, at the MS outpatient clinic of Health Sciences University, Adana City Training and Research Hospital.

A total of 98 patients diagnosed with RRMS and secondary progressive MS (SPMS) according to the 2017 McDonald criteria,^[9]^ along with 40 control patients whose Hcal levels were measured, were included in the study.

#### 2.1.1. Inclusion criteria

1. Patients meeting the 2017 McDonald criteria.
2. Patients aged 18 years and older.
3. Patients who consented to participate in the study.
4. Patients who followed up at our MS outpatient clinic and had no additional comorbidities.

#### 2.1.2. Exclusion criteria

1. Patients under 18 years of age.
2. Patients with conditions that may affect Hcal levels (e.g., systemic inflammatory diseases).
3. Patients who refused to participate in the study.

Of the total 138 patients included in the study, 40 constituted the healthy control (HC) group. Among the remaining patients, 38 were diagnosed with SPMS and 60 with RRMS.

In addition to demographic data, findings on magnetic resonance imaging (cranial MRI), time since initial diagnosis, and number of relapses in the last year were recorded for all patients.

Patients’ expanded disability status scale (EDSS) score, medication use, and MRI lesion load were calculated according to magnetic resonance imaging in multiple sclerosis^[10]^ criteria by a demyelinating diseases specialist. At the time of admission, 10 mL of blood samples were collected from the antecubital vein of all patients, and those samples were centrifuged at 3000 rpm for 5 minutes. The centrifuged blood samples were then analyzed using the enzyme-linked immunosorbent assay method, and the obtained results were recorded in a data form.

### 2.2. Statistical analysis

Statistical analyses were performed using Statistical Package for the Social Sciences 25.0 software (Turkey). Categorical measurements were summarized as counts and percentages, while continuous measurements were presented as means, standard deviations, and minimum-maximum ranges. The Kolmogorov–Smirnov test was used to assess the normality of variable distribution. Chi-square test was employed for comparisons of categorical variables. Non-normally distributed parameters were analyzed using the Mann–Whitney *U* test for 2 groups and the Kruskal–Wallis test for more than 2 groups. The sensitivity and specificity of calprotectin values in predicting the patient group were calculated. Additionally, the area under the receiver operating characteristic curve was analyzed to determine the cutoff value. A statistical significance level of 0.05 was set for all tests.

## 3. Results

A total of 138 patients, with 88 (63.8%) women and 50 (36.2%) men, were included in the study. The average age of the patients was 37.7 ± 9.6 years (median: 36.5). Average height was 1.68 ± 0.08 m (median: 1.67 m), average weight was 71.1 ± 13.1 kg (median: 70 kg), and average body mass index was 25.2 ± 3.8 (median: 24.9). The categories of duration since initial MS diagnosis were distributed as follows: 1 to 4 years for 44 (45.4%), 5 to 10 years for 18 (18.6%), and over 10 years for 35 (36.1%). Regarding MS type, 59 (60.8%) had RRMS, and 38 (39.2%) had SPMS. When the relapses in the past year were evaluated, 26 (26.8%) patients had 1 to 2 attacks; conversely, 71 (73.2%) patients experienced no attacks. DMTs used by the patient group were distributed as follows: 21 (21.6%) received injectable/teriflunomide DMTs, 35 (36.1%) used fingolimod/natalizumab/cladribine, 9 (9.3%) were on natalizumab, and 32 (33%) were treated with ocrelizumab. Cranial MRI lesion distribution in the patient group showed T2 10 lesions in 18 (18.6%), T2 10 to 50 lesions in 60 (61.9%), T2 50 to 100 lesions in 14 (14.4%), and T2 > 100 lesions in 5 (5.2%) patients. Of all the 138 participants, 97 (70.3%) were MS patients, and 41 (29.7%) were in the control group. The average EDSS score of all patients was 2.73 ± 1.7 (median: 2.25), and the average Hcal level was 778.9 ± 336.9 (median: 708) (Table 1).

**Table 1:** Analysis of demographic data of patients.

	Count (n)	Percentage (%)
Gender		
Female	88	63.8
Male	50	36.2
Medical history (n = 97)	29	29.9
Time after MS diagnose (n = 97)		
1–4 yr	44	45.4
5–10 yr	18	18.6
10 yr and above	35	36.1
MS type (n = 97)		
RRMS	59	60.8
SPMS	38	39.2
Relapse in last year (n = 97)		
No relapse	71	73.2
1–2 relapses	26	26.8
DMT agent used (n = 97)		
Inj-teri-dmf	21	21.6
Fingo-nata-clad	35	36.1
Natalizumab	9	9.3
Ocrelizumab	32	33.0
Cranial MRI (n = 97)		
T2-10	18	18.6
T2-10–50	60	61.9
T2-50–100	14	14.4
T2-100 and higher	5	5.2
Study group		
Patients	97	70.3
Control	41	29.7

	Mean ± SD	Med (Min–Max)
Age (yr)	37.7 ± 9.6	36.5 (19–64)
Height (m)	1.68 ± 0.08	1.67 (1.5–1.92)
Weight (kg)	71.1 ± 13.1	70 (45–110)
BMI (kg/m^2^)	25.2 ± 3.8	24.9 (17.6–35.1)
EDSS	2.73 ± 1.7	2.25 (1–6.5)
Human calprotectin (µg/mg)	778.9 ± 336.9	708 (432–3098)
CRP (mg/dL)	3.44 ± 7.6	2 (0.6–74.8)
Albumin (g/dL)	43.2 ± 3.9	43 (27.3–53.1)
Fibrinogen (mg/dL)	3.15 ± 0.8	3.13 (1.6–5.6)

BMI = body mass index, CRP = C-reactive protein, DMT = disease-modifying therapies, EDSS = expanded disability status scale, Fingo-nata-clad = fingolimod-natalizumab-cladribine, Inj-teri-dmf = injectable disease-modifying therapies-teriflunomide-dimethyl fumarate, Max = maximum, Med = median, Min = minimum, MRI = magnetic resonance imaging, MS = multiple sclerosis, RRMS = relapsing-remitting multiple sclerosis, SD = standard deviation, SPMS = secondary progressive multiple sclerosis.

The average age and HCal levels in the patient group were found to be higher compared to the control group (*P* = .013 and *P* < .001, respectively). No significant differences were observed between groups for other parameters presented in Table 2 (*P* > .05). Although the mean fecal calprotectin (Hcal) level was higher in female patients compared to male patients, this difference was not statistically significant (*P* > .05).

**Table 2:** Comparison of demographic and human calprotectin levels of patient and control groups, and between genders.

Gender	Patient (n = 97)	Control (n = 41)	*P* †
	n (%)	n (%)	
Gender			
Female	63 (64.9)	25 (61)	.657
Male	34 (35.1)	16 (39)	

	Mean ± SD (mean)	Mean ± SD (mean)	*P* †
Age (yr)	39.2 ± 10.3 (39)	34.3 ± 6.7 (35)	.013*
Height (m)	1.67 ± 0.08 (1.65)	1.69 ± 0.09 (1.68)	.230
Weight (kg)	70.6 ± 13.0 (70)	72.2 ± 13.3 (72)	.428
BMI (kg/m^2^)	25.2 ± 3.9 (24.9)	25.2 ± 3.5 (25.4)	.708
Human calprotectin (µg/mg)	868.6 ± 357.4 (755)	567 ± 129.3 (530)	<.001**

	Female (n = 88)	Male (n = 50)	*P* ‡
	Mean ± SD (mean)	Mean ± SD (mean)	
Human calprotectin (µg/mg)	798.6 ± 344.5 (719)	744.4 ± 323.6 (687)	.138

BMI = body mass index, ROC = receiver operating characteristic, SD = standard deviation.

† ROC curve test.

‡ Mann–Whitney *U.*

* *P* < .05.

** *P* < .001.

**Figure 1.**
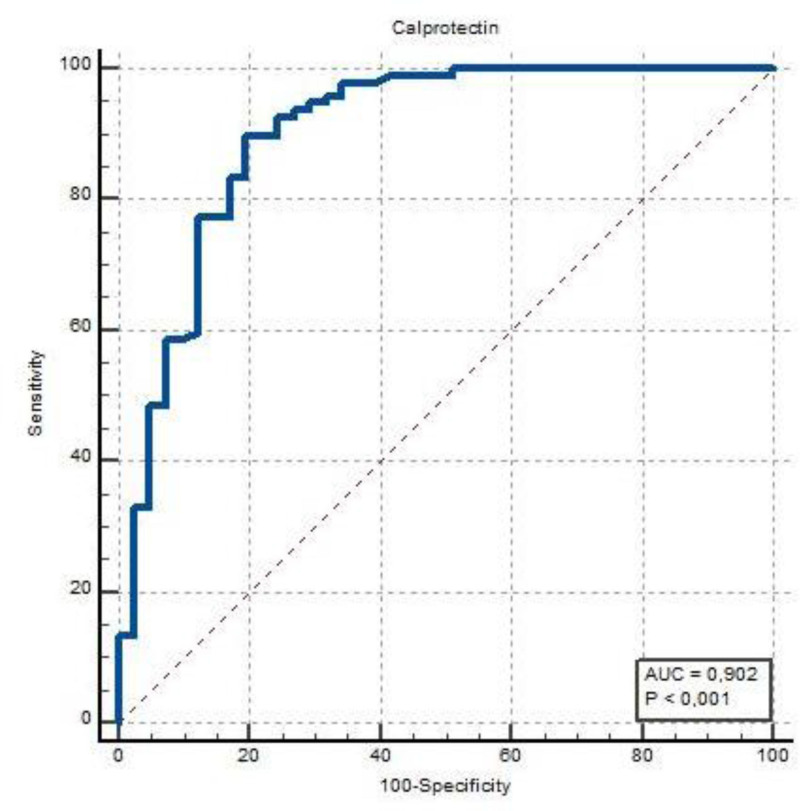
Evaluation of calprotectin values with ROC curve test for patient group prediction. ROC = receiver operating characteristic.

Receiver operating characteristic curve test was performed to determine the predictive ability of calprotectin values for the patient group. The analysis revealed that a calprotectin value above 616 predicted the patient group with a 90.2% area under the receiver operating characteristic curve, 86.69% sensitivity, and 80.49% specificity (Table 3) (Fig. 1). Patients with high calprotectin levels had lower average EDSS scores (*P* = .029). No significant differences were observed between groups for other parameters presented in Table 4 (*P* > .05). A higher frequency of the RRMS type was observed in patients with a high calprotectin cutoff value within the patient group (*P* = .035).

**Table 3:** Evaluation of calprotectin values with the ROC curve test for patient group prediction.

	Human calprotectin
Cutoff	>616
Area under curve (%95 CI)	0.902 (0.840–0.946)
Sensitivity (%95 CI)	89.69 (81.9–94.9)
Specificity (%95 CI)	80.49 (65.1–91.2)
Positive predictive value (%95 CI)	91.6 (85.3–95.3)
Negative predictive value (%95 CI)	76.7 (64.3–85.8)
*P* value	**<.001** **

ROC curve test.

CI = confidence interval , ROC = receiver operating characteristic.

* *P* < .05.

** *P* < .001.

**Table 4:** Analysis of human calprotectin according to the cutoff value.

	Calprotectin (>616)		*P* †
	Low (n = 43)	High (n = 95)	
Gender			
Female	24 (55.8)	64 (67.4)	.191
Male	19 (44.2)	31 (32.6)	
Study group			
Patient	10 (23.3)	87 (91.6)	<.001**
Control	33 (76.7)	8 (8.4)	

	Med ± SD (Med)	Med ± SD (Med)	*P* ‡
Age (yr)	35.2 ± 7.8 (35)	38.9 ± 10.2 (38)	.052
Height (m)	1.69 ± 0.08 (1.69)	1.67 ± 0.09 (1.65)	.178
Weight (kg)	71.7 ± 13.3 (72)	70.8 ± 13.0 (70)	.575
BMI (kg/m^2^)	25.1 ± 3.7 (25.4)	25.3 ± 3.9 (24.8)	.901
Human calprotectin (µg/mg)	3.9 ± 1.9 (4.75)	2.60 ± 1.6 (2)	**.029** *

BMI = body mass index, Med = median, SD = standard deviation.

† Chi-square.

‡ Mann–Whitney *U*.

* *P* < .05.

Patients in the patient group with a high calprotectin cutoff value also had lower average EDSS scores (*P* = .029). No significant differences were found among other parameters in Table 5 (*P* > .05). The proportion of patients with high Hcal cutoff values was significantly greater in both the RRMS and SPMS groups compared to the control group (*P* < .001) (Table 6).

**Table 5:** Analysis of human calprotectin according to the cutoff value in the patient group.

	Calprotectin (>616)		*P* †
	Low (n = 10)	High (n = 87)	
Gender			
Female	4 (40)	59 (67.8)	.081
Male	6 (60)	28 (32.2)	
Medical history (n = 97)	1 (10)	28 (32.2)	.147
Time after MS diagnose (n = 97)			
1–4 yr	4 (40)	40 (46)	.615
5–10 yr	3 (30)	15 (17.2)	
10 yr and above	3 (30)	32 (36.8)	
MS type (n = 97)			
RRMS	3 (30)	56 (64.4)	**.035** *
SPMS	7 (70)	31 (35.6)	
Relapse in last year (n = 97)			
No relapse	8 (80)	63 (72.4)	.608
1–2 relapses	2 (20)	24 (27.6)	
DMT agent used (n = 97)			
Inj-teri-dmf	1 (10)	20 (23)	.230
Fingo-nata-clad	3 (30)	32 (36.8)	
Natalizumab	–	9 (10.3)	
Ocrelizumab	6 (60)	26 (29.9)	
Cranial MRI (n = 97)			
T2-10	–	18 (20.7)	.053
T2-10–50	6 (60)	54 (62.1)	
T2-50–100	4 (40)	10 (11.5)	
T2-100 and higher	–	5 (5.7)	

	Mean ± SD (Med)	Mean ± SD (Med)	*P* ‡
Age (yr)	38.4 ± 9.9 (37.5)	39.3 ± 10.4 (39)	.803
Height (m)	1.69 ± 0.08 (1.71)	1.67 ± 0.08 (1.65)	.409
Weight (kg)	69.3 ± 15.2 (66.5)	70.7 ± 12.9 (70)	.717
BMI (kg/m^2^)	24.1 ± 3.9 (24.0)	25.3 ± 3.9 (24.9)	.406
EDSS	3.90 ± 1.9 (4.75)	2.60 ± 1.6 (2)	**.029** *
Human calprotectin (µg/mg)	2.95 ± 2.3 (2)	3.53 ± 8.1 (2)	.728
CRP (mg/dL)	43.4 ± 3.8 (43.5)	43.1 ± 3.9 (43)	.452
Albumin (g/dL)	2.68 ± 0.7 (2.63)	3.20 ± 0.8 (3.17)	.051

BMI = body mass index, CRP = C-reactive protein, DMT = disease-modifying therapies, EDSS = expanded disability status scale, Fingo-nata-clad = fingolimod-natalizumab-cladribine, Inj-teri-dmf = injectable disease-modifying therapies-teriflunomide-dimethyl fumarate, Med = median, MRI = magnetic resonance imaging, MS = multiple sclerosis, RRMS = relapsing-remitting multiple sclerosis, SD = standard deviation, SPMS = secondary progressive multiple sclerosis.

† Chi-square.

‡ Mann–Whitney *U.*

* *P* < .05.

**Table 6:** Analysis of multiple sclerosis subtypes and the control group according to the human calprotectin cutoff value.

	Control (n = 41)	RRMS (n = 59)	Secondary progressive MS (n = 38)	*P* †
	n (%)	n (%)	n (%)	
Gender				
Female	25 (61)	42 (71.2)	21 (55.3)	.255
Male	16 (39)	17 (28.8)	17 (44.7)	
Medical history (n = 97)	–	16 (27.1)	13 (34.2)	.456
Time after MS diagnose (n = 97)				
1–4 yr	–	32 (54.2)	12 (31.6)	.051
5–10 yr	–	11 (18.6)	7 (18.4)	
10 yr and above	–	16 (27.1)	19 (50)	
Relapse in last year (n = 97)				
No relapse	–	42 (71.2)	29 (76.3)	.578
1–2 relapses	–	17 (28.8)	9 (23.7)	
DMT agent used (n = 97)				
Inj-teri-dmf	–	21 (35.6)	-	**<.001** **
Fingo-nata-clad	–	31 (52.5)	4 (10.5)	
Natalizumab	–	5 (8.5)	4 (10.5)	
Ocrelizumab	–	2 (3.4)	30 (78.9)	
Cranial MRI (n = 97)				
T2-10	–	11 (18.6)	7 (18.4)	.300
T2-10–50	–	40 (67.8)	20 (52.6)	
T2-50–100	–	6 (10.2)	8 (21.1)	
T2-100 over	–	2 (3.4)	3 (7.9)	
Human calprotectin cutoff				
Low	33 (80.5)	3 (5.1)	7 (18.4)	**<.001** **
High	8 (19.5)	56 (94.9)	31 (81.6)	

	Mean ± SD (Med)	Mean ± SD (Med)	Mean ± SD (Med)	*P*
Age (yr)	34.3 ± 6.7 (35)	34.9 ± 9.3 (32)	45.8 ± 8.5 (44.5)	<.001**,§
Height (m)	1.69 ± 0.09 (1.68)	1.67 ± 0.08 (1.65)	1.67 ± 0.08 (1.66)	.483§
Weight (kg)	72.2 ± 13.3 (72)	71.1 ± 13.9 (70)	69.8 ± 11.6 (69)	.668§
BMI (kg/m^2^)	25.2 ± 3.5 (25.4)	25.4 ± 4.3 (24.9)	24.9 ± 3.4 (24.7)	.880§
EDSS	–	1.69 ± 1.07 (1)	4.35 ± 1.2 (4.5)	<.001**,‡
Human calprotectin (µg/mg)	–	2.84 ± 2.7 (2)	4.44 ± 11.8 (2.05)	.378‡
CRP (mg/dL)	–	43.5 ± 4.3 (43)	42.6 ± 3.3 (43)	.449‡
Albumin (g/dL)	–	2.98 ± 0.7 (3.08)	3.41 ± 0.9 (3.46)	.025*,‡

BMI = body mass index, CRP = C-reactive protein, DMT = disease-modifying therapies, EDSS = expanded disability status scale, Fingo-nata-clad = fingolimod-natalizumab-cladribine, Inj-teri-dmf = injectable disease-modifying therapies-teriflunomide-dimethyl fumarate, Med = median, MRI = magnetic resonance imaging, MS = multiple sclerosis, SD = standard deviation.

† Chi-square.

‡ Mann–Whitney *U*.

§ Kruskal–Wallis test.

* *P* < .05.

** *P* < .001.

## 4. Discussion

MS is a complex autoimmune disease targeting the CNS, characterized by inflammation, demyelination, and neurodegeneration. The clinical presentation of MS is variable since patients experience diverse patterns of relapse activity and disability progression; it is often challenging to discern various types and courses of the disease.^[11]^ Currently, MS diagnosis relies on a combination of clinical observations, patient medical history, physical examination, findings from MRI, and analysis of CSF for oligoclonal bands after performing an invasive lumbar puncture. A significant challenge also exists in differentiating between the various MS subtypes, such as RRMS and the progressive forms, due to overlapping clinical presentations and radiological features.^[12]^ This underlines a pressing need for reliable biomarkers that can be utilized in earlier diagnosis, predict disease course, monitor disease activity, and accurately distinguish between subtypes. Unfortunately, very few studies have moved beyond the research and validation phase and been successfully implemented in patient care. A good MS biomarker should be easily measurable, correlate with disease-related pathophysiology, such as inflammatory activity, neurodegeneration and demyelination.^[13]^ The reduced invasiveness of blood sampling makes serum-based biomarkers preferred options when available, allowing for easy and frequent measurements compared to the collection of CSF. In light of these unmet diagnostic and prognostic demands, we evaluated serum calprotectin (S100A8/A9) values for its potential as a biomarker in MS. Calprotectin is a calcium-binding complex protein composed of subunits of myeloid-related protein 8 (S100A8) and 14 (S100A9), also known as calgranulin A and B.^[14]^ Calprotectin plays an active role in several key cellular processes related to inflammation, including influencing kinase activities and guiding the differentiation and movement of monocytes and microglia across endothelial barriers.^[15]^ Elevated serum levels of calprotectin have been previously reported in several inflammatory conditions, including rheumatoid arthritis, systemic lupus erythematosus, and IBD.^[16]^ However, there are only a few studies investigating the efficacy of serum calprotectin as a predictive biomarker in MS, and none have evaluated its ability to differentiate between various MS types and courses. Given calprotectin’s established role as an indicator of inflammation in other contexts, our study investigated its potential as a serum biomarker for diagnosing MS, demonstrating significantly higher levels in MS patients than the HC group and further suggesting an association between elevated calprotectin and the relapsing-remitting subtype.

Serum calprotectin demonstrates promising diagnostic utility in MS according to various studies. In the study by Olsson et al, serum calprotectin levels were analyzed in 49 newly diagnosed, untreated RRMS patients and 58 HCs. These findings were later confirmed in a validation cohort comprising 68 newly diagnosed, treatment-naive RRMS patients and 50 HCs. The results consistently showed that patients with active MS had significantly higher serum calprotectin levels compared to those with inactive MS and HCs.^[17]^ Similarly, in our study, MS patients exhibited significantly higher average calprotectin levels (868.6 ± 357.4) compared to the disease-free control group (567 ± 129.3) (*P* < .001), and calprotectin levels above 616 could predict the patient group with 90.2% accuracy (95% confidence interval: 0.840–0.946). Supporting our findings, another study similarly reported elevated calprotectin levels in MS patients: in 28 patients with relapsing MS, median serum MRP-8/14 levels were significantly higher (5150 ng/mL) compared to 26 HCs (1482 ng/mL), and notably higher during acute relapse (6690 ng/mL) versus stable disease (3050 ng/mL), suggesting early macrophage activation in demyelinating plaques.^[18]^ Additionally, while the study of Nouri et al primarily focused on fecal levels of the biomarker, it revealed an early increase in plasma calprotectin with concentrations nearly doubling by day 7 postimmunization before symptom onset, and tripled by day 21 compared to HCs, suggesting that changes in calprotectin concentrations occur early in disease development.^[19]^ Calprotectin has also been observed to be elevated in the CSF of a significant proportion of MS patients. CSF calprotectin levels are suggested to be higher in the early stages of the disease, within 2 weeks of symptom onset, compared to later stages.^[4]^ These results imply that the presence of phagocytosing cells in active CNS lesions, even in early stages, suggests that CSF calprotectin could be a particularly useful marker for early diagnosis. It is also important to consider that calprotectin values vary with age. In healthy individuals, fecal calprotectin concentrations can range from 0 to 545.9 µg/g, exhibiting an age-related difference.^[20]^ This suggests that age-specific cutoff values for calprotectin may be necessary. In our study, the patient cohort’s average age was 39.2 ± 10.3 years, which was significantly higher than that of the control group (*P* = .013). This age disparity is relevant given the association between increasing age and higher calprotectin levels. Therefore, conclusions regarding biomarker levels and disease progression in our study should be interpreted acknowledging age as a potential confounding factor. Still, collective findings of previous studies align with our own results and point to increased systemic inflammation and myeloid cell activation as key features of MS, positioning serum calprotectin as a promising and accessible biomarker in MS diagnosis and activity monitoring.

MS is clinically categorized into several subtypes, the most common being the RRMS, which is characterized by unpredictable relapses followed by remission. Progressive forms, including PPMS and SPMS, involve a gradual accumulation of neurological disability. Accurate differentiation between these subtypes is critical for prognosis and managing treatment options.^[21]^ Numerous immunological studies were conducted to find and identify MS-specific biomarkers capable of predicting clinical disease course and outcome.^[22]^ Previous studies have involved a comprehensive examination of various inflammatory mediators like cytokines, chemokines, and adhesion molecules, as well as detailed immunophenotyping of inflammatory cells in the CSF. These analyses have revealed increased concentrations of these substances in the serum and CSF of MS patients, with the highest increase mostly seen in patients with active RRMS, and to a lesser extent in those with SPMS and PPMS.^[23,24]^ While these markers offer some clinical value for diagnosis and monitoring disease activity, none have proven to be specific for MS. We propose calprotectin as a potential serum biomarker for differentiating between RRMS and SPMS based on our findings: within the patient group, a higher frequency of RRMS was observed in patients with a high calprotectin cutoff value (64.4% vs 30% in low-calprotectin group) (*P* = .035). Meanwhile, SPMS was more prevalent in patients with lower calprotectin levels (70% vs 35.6% in the high calprotectin group), suggesting that increased calprotectin levels may indicate a relapsing-remitting phenotype of MS. This observed association between elevated calprotectin levels and the relapsing-remitting phenotype most likely originates from the increased inflammatory activity experienced during relapse episodes. It is theorized that MS begins with a prominent inflammatory phase typical of RRMS, but then transitions to a progressive stage (SPMS) where neurodegeneration, often independent from acute inflammation, becomes the primary mechanism of disability progression.^[12]^ While brain and spinal cord inflammation are present not only in RRMS but also in SPMS, the extent of CNS inflammation also declines with age and disease duration. However, the literature research presents a more complex picture regarding serum calprotectin’s effectiveness as a standalone biomarker. A systematic review specifically aimed at identifying biomarkers that could differentiate between RRMS and SPMS found that while several biomarkers, including neurofilament light chain and glial fibrillary acidic protein, showed promising results, the evidence for the efficiency of calprotectin in this context was less consistent and based on a limited number of studies.^[25]^ The review cautions against the clinical regular use of calprotectin levels to distinguish between RRMS and progressive MS, suggesting that observed elevations in calprotectin appear to be more related to the overall inflammatory state and disease activity rather than the specific clinical course of the disease. MS mainly affects the CNS, and growing evidence points to systemic immune dysfunction as one of the main sources of elevated calprotectin levels, as seen in other inflammatory conditions. Calprotectin is known for its high sensitivity to intestinal inflammation in IBD, though its specificity is debatable. Similarly, in MS, while low calprotectin could help exclude active inflammation, elevated readings might stem from other causes of inflammation. It is important to remember that even in the progressive stage of the disease, pronounced inflammation is present, and this inflammatory burden can be as high as that in other acute or chronic inflammatory conditions, vastly exceeding levels seen in metabolic or neurodegenerative illnesses.^[26]^ Although our findings suggest calprotectin’s potential as a diagnostic marker for MS with promising accuracy, sensitivity, and specificity, it is important to consider that elevated calprotectin levels are not exclusive to MS and can be observed in other neurological conditions, with significantly higher serum calprotectin levels reported in patients with other chronic inflammatory demyelinating conditions in various studies.^[27]^ This may suggest calprotectin could be utilized as a component of a broader diagnostic panel rather than further studies demonstrate its clinical validity as a singular diagnostic indicator for MS.

While direct research on the efficiency of calprotectin in differentiating between these 2 MS courses is limited, studies involving calprotectin often utilize disease activity as a criterion. Active MS is predominantly characterized by clinical relapses of acute exacerbations of neurological symptoms, along with the presence of inflammatory lesions detected on MRI. It is important to note that the specific definition of “disease activity” is not a uniform definition and can vary across research studies. Still, the presence of active disease is often associated with the relapsing stages of the relapsing-remitting form of MS, and therefore holds value in terms of being more closely linked to the RRMS than progressive forms that involve disease progression without such acute inflammatory events. Additionally, since the activation of microglia and infiltration of macrophages are characteristic features of active MS lesions regardless of how long the disease has been present, and the MRP14 subunit of calprotectin is selectively expressed by macrophages within these active lesions, calprotectin can be considered as a potential biomarker for disease activity and the relapsing phase of MS. In a study of 211 patients with MS and various other neurological diseases, Berg-Hansen et al analyzed calprotectin levels in CSF and serum and stated that while CSF calprotectin concentration increases during acute neuroinflammation and reflects disease activity in patients with MS and clinically isolated syndrome, serum calprotectin did not reflect disease activity in MS or clinically isolated syndrome.^[28]^ The neuroinflammatory process in MS is often compartmentalized by the blood-brain barrier, which may prevent its systemic reflection. Still, focal disruption of the blood-brain barrier is a hallmark of MS and the substantial calprotectin gradient between serum and CSF means that even minor breaches could release CNS-derived calprotectin into the peripheral circulation. This may explain the observed elevated serum/plasma levels in our study, and specifically the higher incidence of RRMS among patients with high serum calprotectin. In contrast to our findings, the study of Floris et al, conducted with 16 RRMS patients to investigate whether serum levels of MRP-8/14 complexes correlated with disease activity, concluded that calprotectin serum levels may not reflect disease activity in RRMS. Despite observing large variations in serum MRP-8/14 levels, their findings revealed that levels were not related to changes in clinical disease activity or increases in Gd-DTPA lesion enhancement.^[29]^ However, Nouri et al reported results similar to ours, with plasma calprotectin concentrations rising significantly (up to 3-fold) by day 21 compared to HCs. Their data indicated a strong correlation between increasing calprotectin levels, disease progression, disease activity, and the presence of phagocytosing cells in inactive CNS lesions. These elevated levels of calprotectin during the relapsing phase of MS patients were considered to reflect the underlying neuroinflammatory processes characteristic of the disease.^[18]^ CD4+ T-cells trigger the initial immune response in MS, yet B-cells, CD20+ variants and plasma cells are the main culprits of brain inflammation, demyelination, and neurodegeneration.^[26]^ The prominent role of B-cells in inflammatory lesions aligns with the characteristic relapses and remissions of RRMS, where periods of high disease activity are driven by adaptive immune responses involving B-cell lineage cells within the CNS.^[30]^

Interestingly, our study revealed an inverse relationship between calprotectin levels and disability, as measured by the EDSS. Patients with higher calprotectin values had a lower average EDSS score (2.60 ± 1.6) compared to those with lower calprotectin values (3.9 ± 1.9, *P* = .029). This finding might seem counterintuitive, since higher inflammation, as indicated by elevated calprotectin, is generally associated with disease activity in MS, but not with acquired disabilities. It is possible that during the earlier stages of the disease, which are characterized by more inflammatory relapses and potentially higher calprotectin, the overall disability might be lower compared to later progressive stages, where inflammation might be lower but neurodegeneration becomes more prominent, leading to higher EDSS scores. The observed inverse relationship between calprotectin levels and EDSS scores might also be directly influenced by disease duration. Our data indicate that patients with lower calprotectin levels, who consequently presented with higher EDSS scores, tended to have a longer duration of MS, with 40% of the low-calprotectin group having 1 to 4 years of disease duration versus 46% in the high calprotectin group.

## 5. Limitations

Our study presents several limitations. Firstly, the relatively small sample size, particularly within the low-calprotectin group, may impact the ability to draw definitive conclusions regarding various DMTs on calprotectin levels and MS outcomes. This challenge is further complicated by the variability in DMT usage across our patient cohort, and may impact the isolation of calprotectin’s independent role as a biomarker. The absence of an HC group may also limit the precise interpretation of MS-specific baseline calprotectin levels.

## 6. Conclusion

Our study suggests the potential of serum calprotectin as a valuable biomarker in MS, both in aiding diagnosis and potentially distinguishing MS subtypes. While our findings indicate a possible association between elevated calprotectin and the relapsing-remitting subtype, it is important to acknowledge that existing literature presents some contradicting evidence regarding its ability to serve as a marker for subtype differentiation. Examining the relationship between calprotectin and the inflammatory and progressive phases of MS can provide important clues about how this biomarker plays a role in the course of MS. Ultimately, improved comprehension of Hcal can guide the development of more personalized MS treatment strategies. Further research and validation studies are needed to fully understand this novel biomarker’s potential and how it can best be used in patient care.

## Author contributions

**Conceptualization:** Elif Banu Soker, Derya Ozdogru, Yusuf Tamam.

**Funding acquisition:** Yusuf Tamam.

**Methodology:** Elif Banu Soker, Yusuf Tamam.

**Software:** Yusuf Tamam.

**Supervision:** Elif Banu Soker, Miray Erdem, Derya Ozdogru, Yusuf Tamam.

**Data curation:** Elif Banu Soker, Miray Erdem.

**Formal analysis:** Elif Banu Soker, Miray Erdem, Derya Ozdogru, Yusuf Tamam.

**Resources:** Elif Banu Soker, Yusuf Tamam.

**Visualization:** Elif Banu Soker, Miray Erdem, Yusuf Tamam.

**Investigation:** Miray Erdem, Derya Ozdogru.

**Project administration:** Miray Erdem, Derya Ozdogru.

**Validation:** Derya Ozdogru.

**Writing – original draft:** Elif Banu Soker, Miray Erdem, Derya Ozdogru.

**Writing – review & editing:** Elif Banu Soker, Derya Ozdogru, Yusuf Tamam.
